# Supplementary material for: RNA atlas of human bacterial pathogens uncovers stress dynamics linked to infection
Source: Nat Commun. 2021 Jun 2;12:3282. doi: 10.1038/s41467-021-23588-w (PMC8172932; doi:10.1038/s41467-021-23588-w)
Supplement: Supplementary file 1 — Supplementary Information [file 41467_2021_23588_MOESM1_ESM.pdf]

RNA atlas of human bacterial pathogens uncovers stress dynamics linked to infection

Kemal Avican[1*], Jehad Aldahdooh[2,3], Matteo Togninalli[4,5], A K M Firoj Mahmud[1],
Jing Tang[2,3], Karsten M. Borgwardt[4,5], Mikael Rhen[6] and Maria Fällman[1*]

Supplementary Information

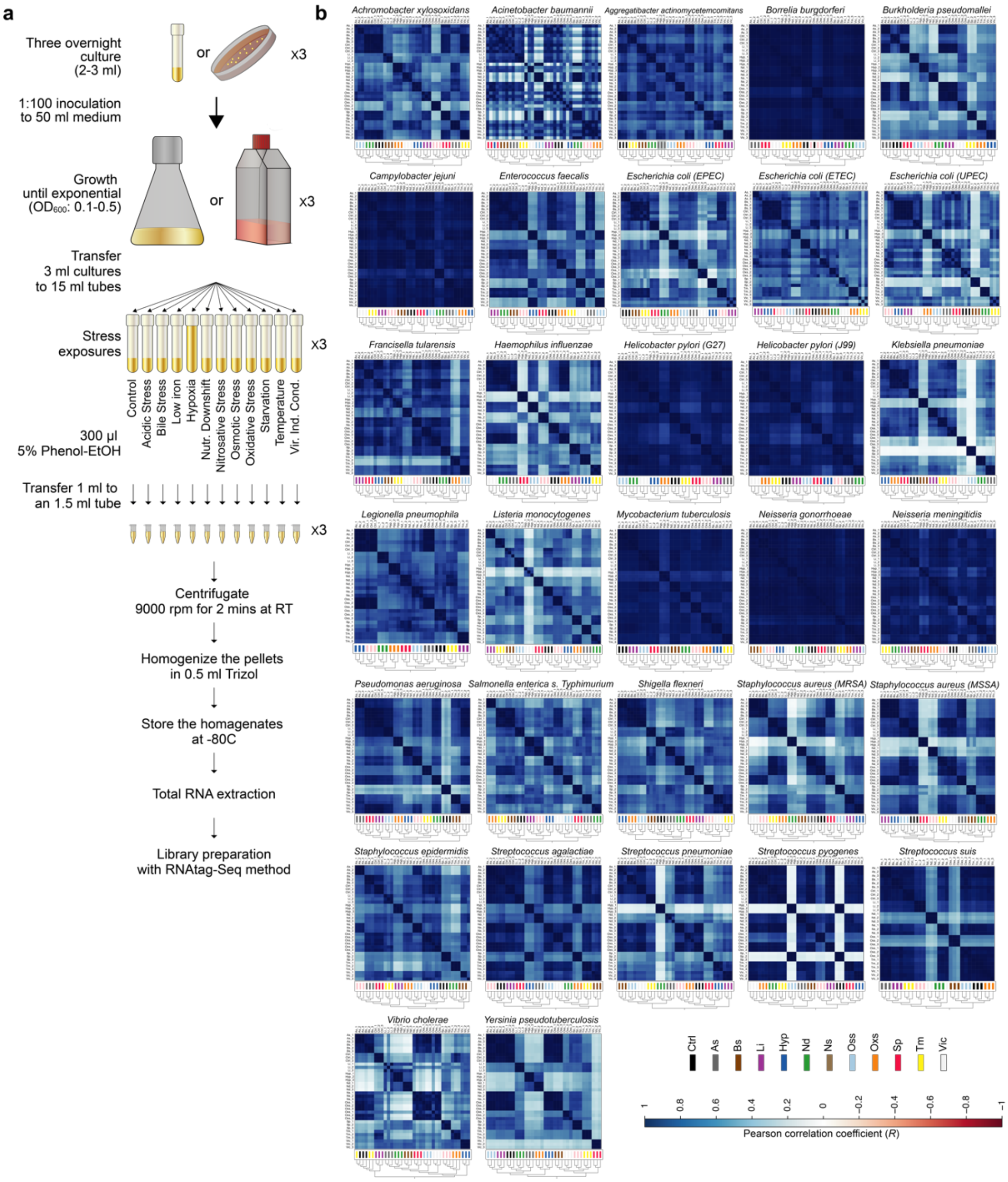

Supplementary Fig. 1 Experimental set-up for stress exposures and global expression pattern of biological replicates. **a**, For the stress exposure experiments, for each strain, three bacterial cultures were inoculated from bacteria grown at stationary phase. Cultures, except control samples, were exposed to the stress conditions at exponential growth (OD_{600} 0.1-0.5). Transcription was stopped by adding 0.05% (final concentration) phenol:ethanol solution. Cells were pelleted and homogenized in Trizol solution and stored at -80 °C until RNA extraction step. The libraries were generated with RNAtag-Seq from total RNA. The specific culturing and stress conditions for each species are indicated in **Supplementary Data 1**. *L. pneumophila* and *M. tuberculosis* were not exposed to the virulence inducing condition, since no such condition has been described. **b**, Correlation coefficients (*R*) of the global expression levels were plotted on a matrix and a dendrogram showing the clustering of replicates with complete linkage (below) for each species. *R* values were calculated by the Pearson method. The matrices were generated using the “corrplot” R package and complete linkage by the CLC Genomics Workbench.

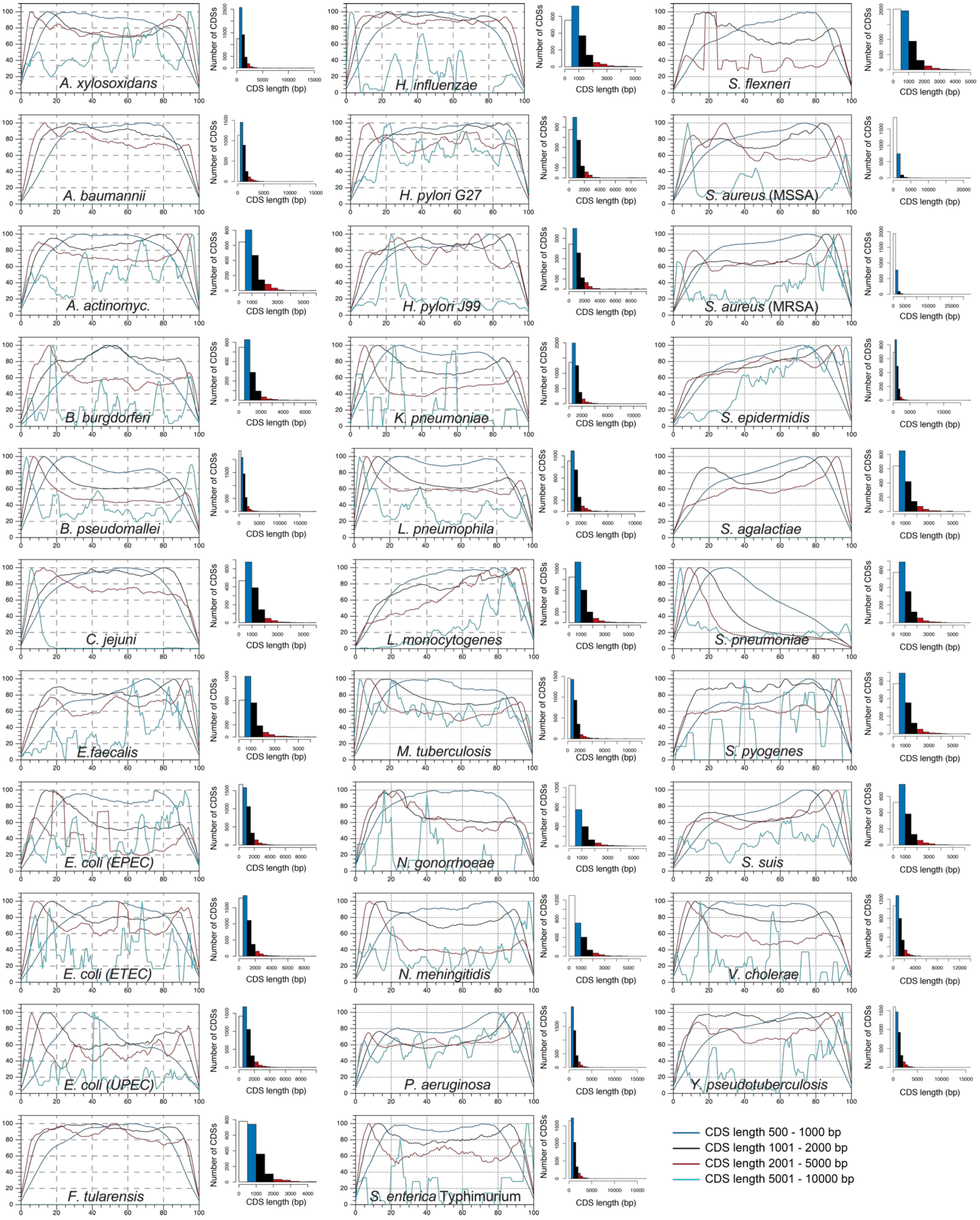

Supplementary Fig. 2. Quality of read mappings through transcript coverage along the CDSs length. The plots show normalized read coverage along the length of different CDSs groups. y-axis indicates normalized number of counts and x-axis indicates normalized length of CDSs grouping in base pair. The CDSs are grouped into 4 groups according to their size (indicated at lower right). The alignments of one sequencing library is presented for each species together with a histogram indicating number of CDSs in each CDSs length groupings. For this analysis the library with the least number of aligned reads were used for each strain.

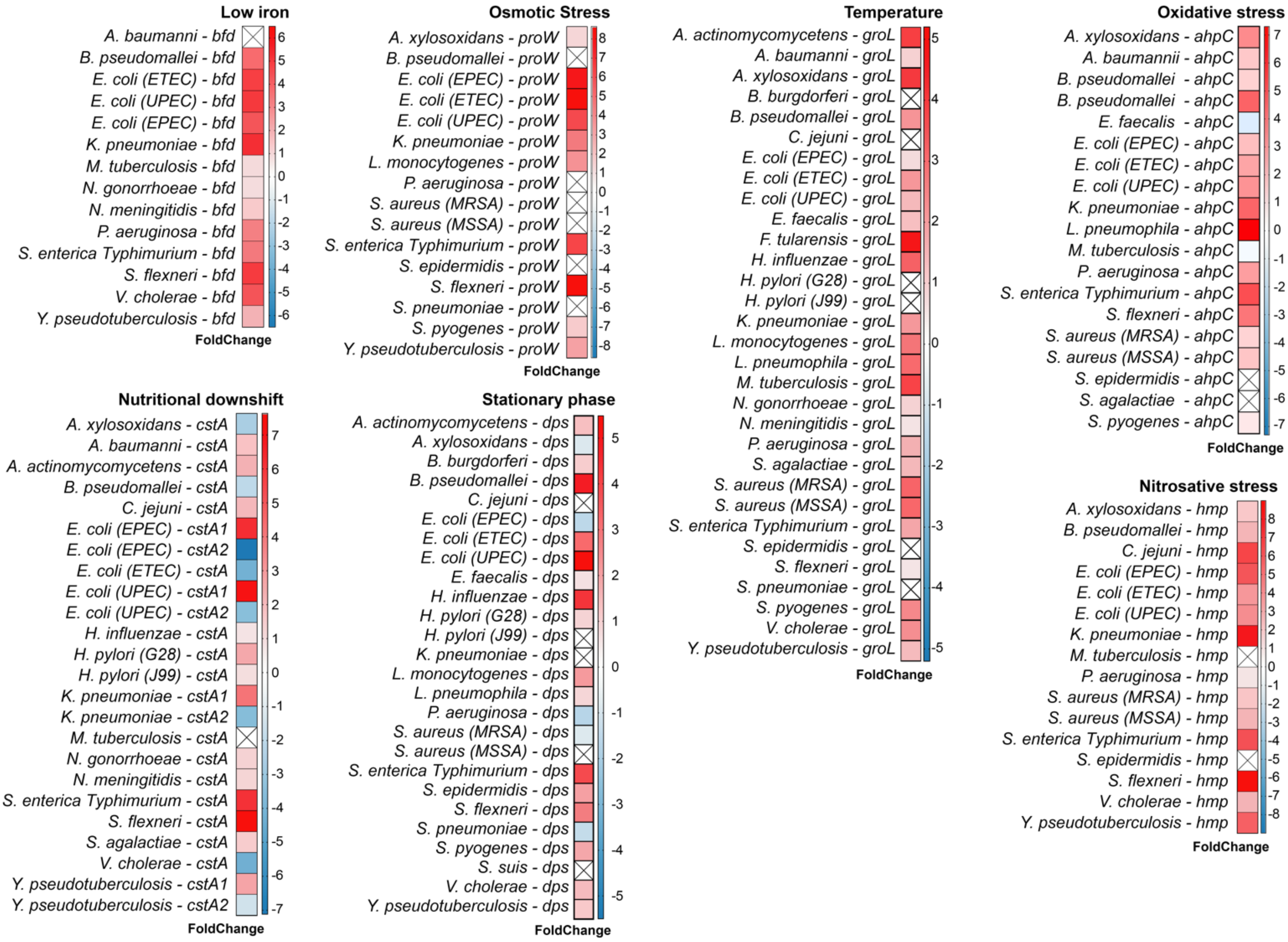

Supplementary Fig. 3. Differential expression of genes previously known to be regulated under particular stress conditions. Differential expression of genes known to be regulated under linked stress condition The differential expression are shown as log2 fold change with FDR-corrected *p-value* < 0.05. Crossed squares indicate genes with no differential regulation under indicated stress condition. Source data are provided as a Source Data file.

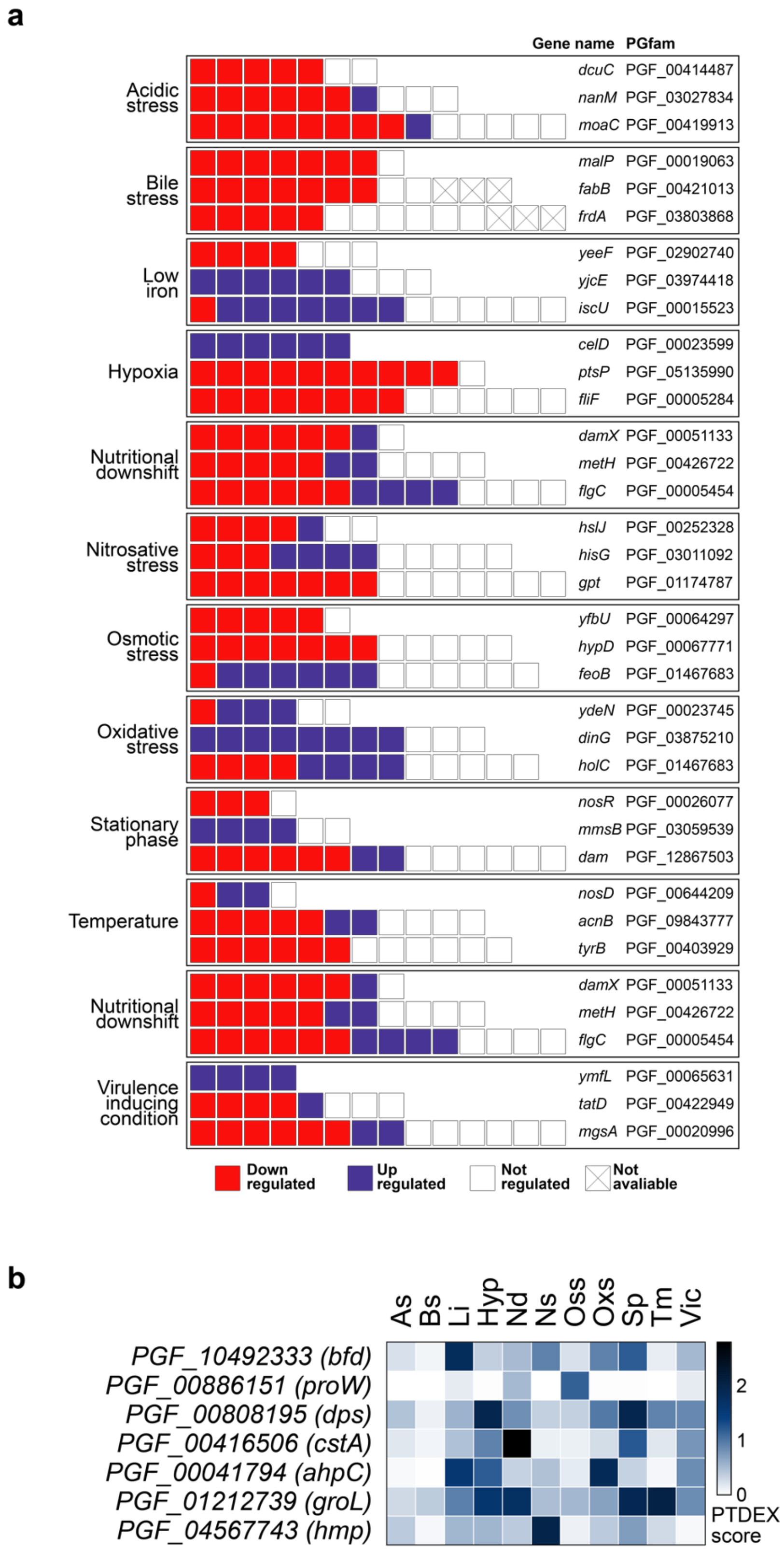

Supplementary Fig. 4. Gene groups with PTDEX score ≥0.25 is accurate for indicating the probability of gene groups being regulated under a particular stress condition and PTDEX scores of genes shown in Supplemental Fig. 3. a, Randomly-picked three PGFam groups whose PTDEX scores were between 0.25-0.3 for each stress condition from Gram-negative strains. One representative gene group of different sizes (groups with 6-7, 8-10, and 12-14 genes) were picked for each stress condition. Each box represents an individual gene from the gene group. Red indicates downregulation, blue indicates upregulation, white indicates not regulated, and crossed white indicates a gene whose expression could not be measured due to lack of RNA. **b,** PTDEX score of genes, shown in Supplementary Fig. 3, previously known to be regulated in a particular condition. Source data are provided as a Source Data file.

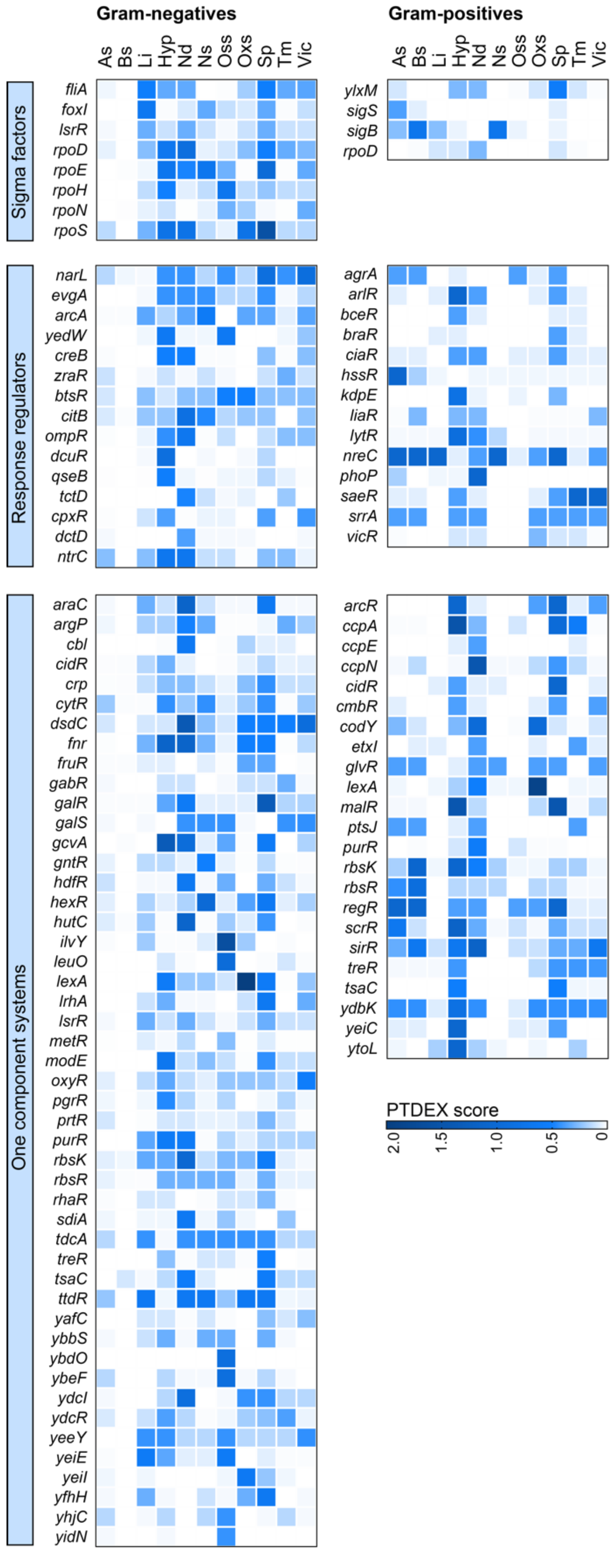

Supplementary Fig. 5. Diverse transcription factors from Gram-negative and -positive bacteria show distinct PTDEX score patterns. PTDEX scores of sigma factors, response regulators, and one-component systems in both Gram-negative and -positive strains. Transcription factors in the different bacteria were identified using the P2TF database (Ortet et al., 2012). Source data are provided as a Source Data file.

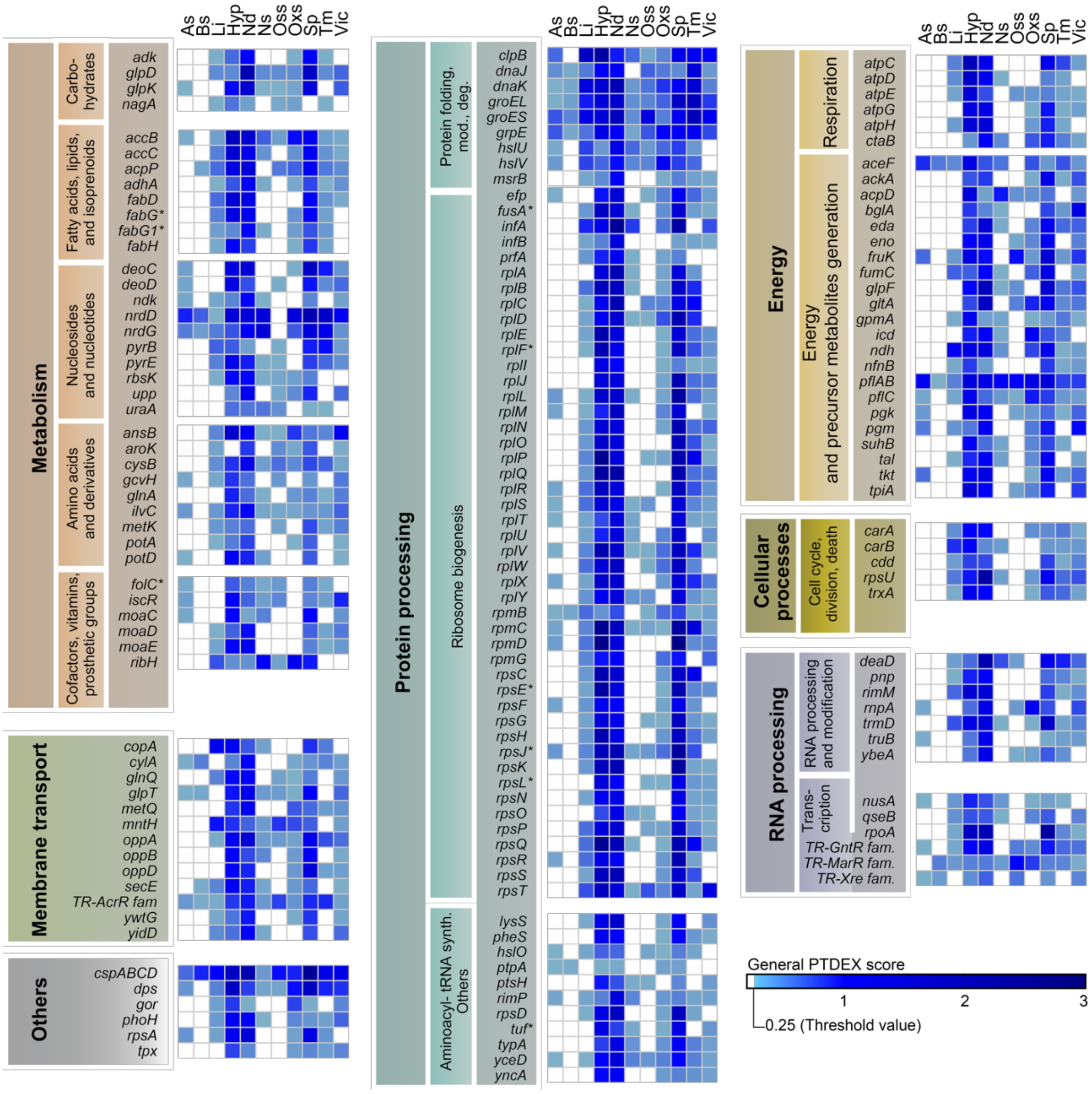

Supplementary Fig. 6. PTDEX scores identify universal stress responder (USR) genes involved in responses to multiple stresses in both Gram-negative and -positive strains. USRs were clustered in different biological processes using the PATRIC Subsystems tool and are shown with their general PTDEX scores over the 11 stress conditions. The USRs which are previously shown to be targets for antibiotics are marked with asterisk. Source data are provided as a Source Data file.

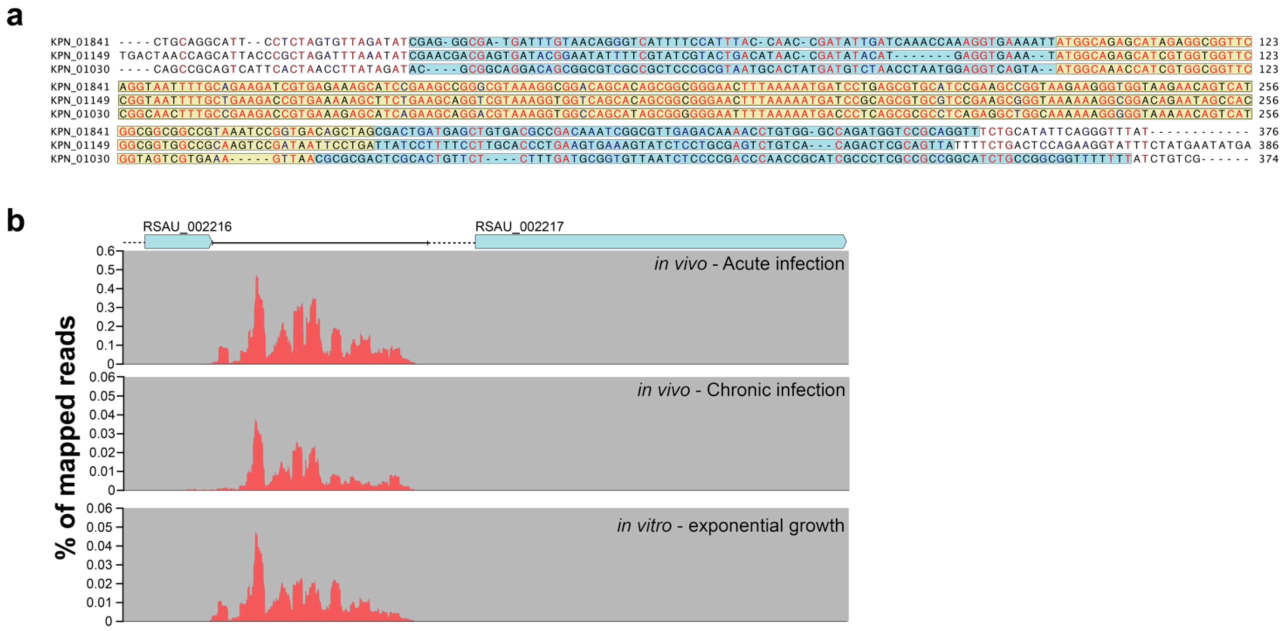

Supplementary Fig. 7. Sequence homology of KPN_01149 paralogs in *K. pneumoniae* and expression of SRS42 in *S. aureus* from *in vivo* samples. a, Multiple sequence alignments of KPN_01149 and its paralogs in *K. pneumoniae*. Sequences marked with blue indicate the 5'-UTR and 3'-UTR. Red-colored nucleotides indicate identical nucleotides among the three sequences. **b,** Reads mapped to SRS42 in *S. aureus* 6850 during acute infection and chronic infection in a murine infection model, and *in vitro* exponential growth (Szafranka et al., 2014).

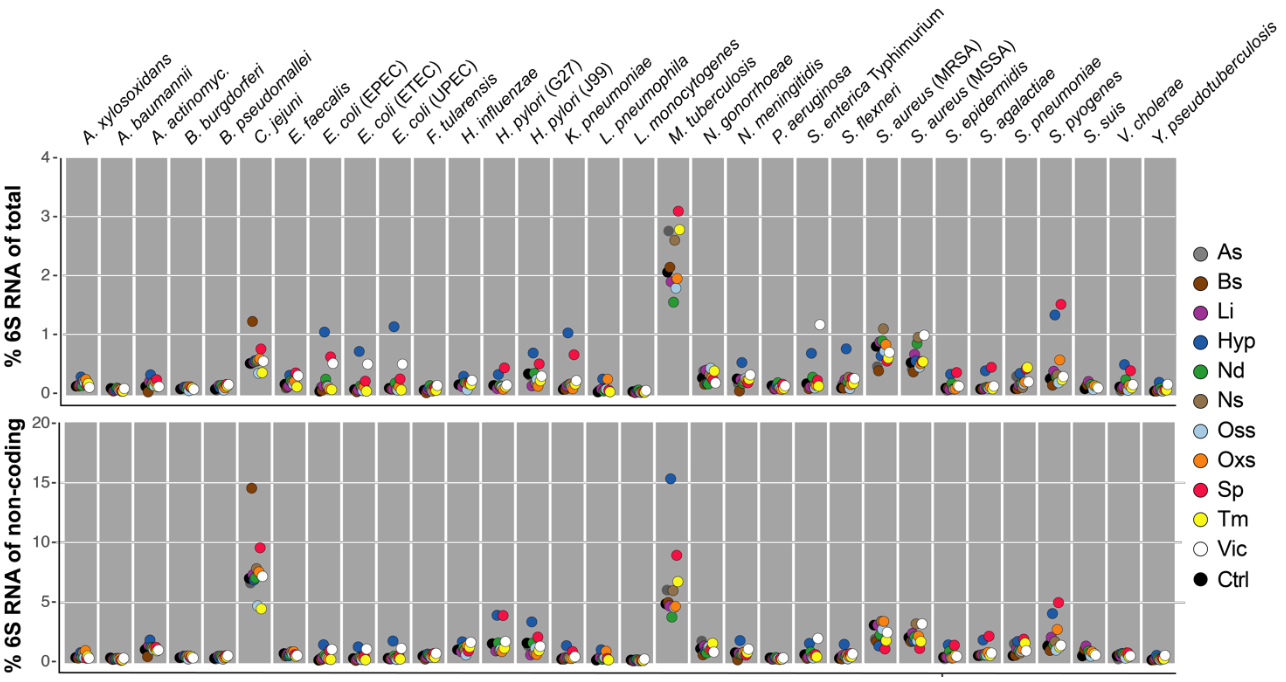

Supplementary Fig. 8. The expression of 6S RNA slightly differs under certain stress conditions. Proportion of reads mapped to 6S RNA of total number of mapped reads to whole genome under each stress condition (upper panel) and proportion of reads mapped to 6S RNA of total number of reads mapped to non-CDSs under each stress condition (lower panel) for all tested strains. Source data are provided as a Source Data file.

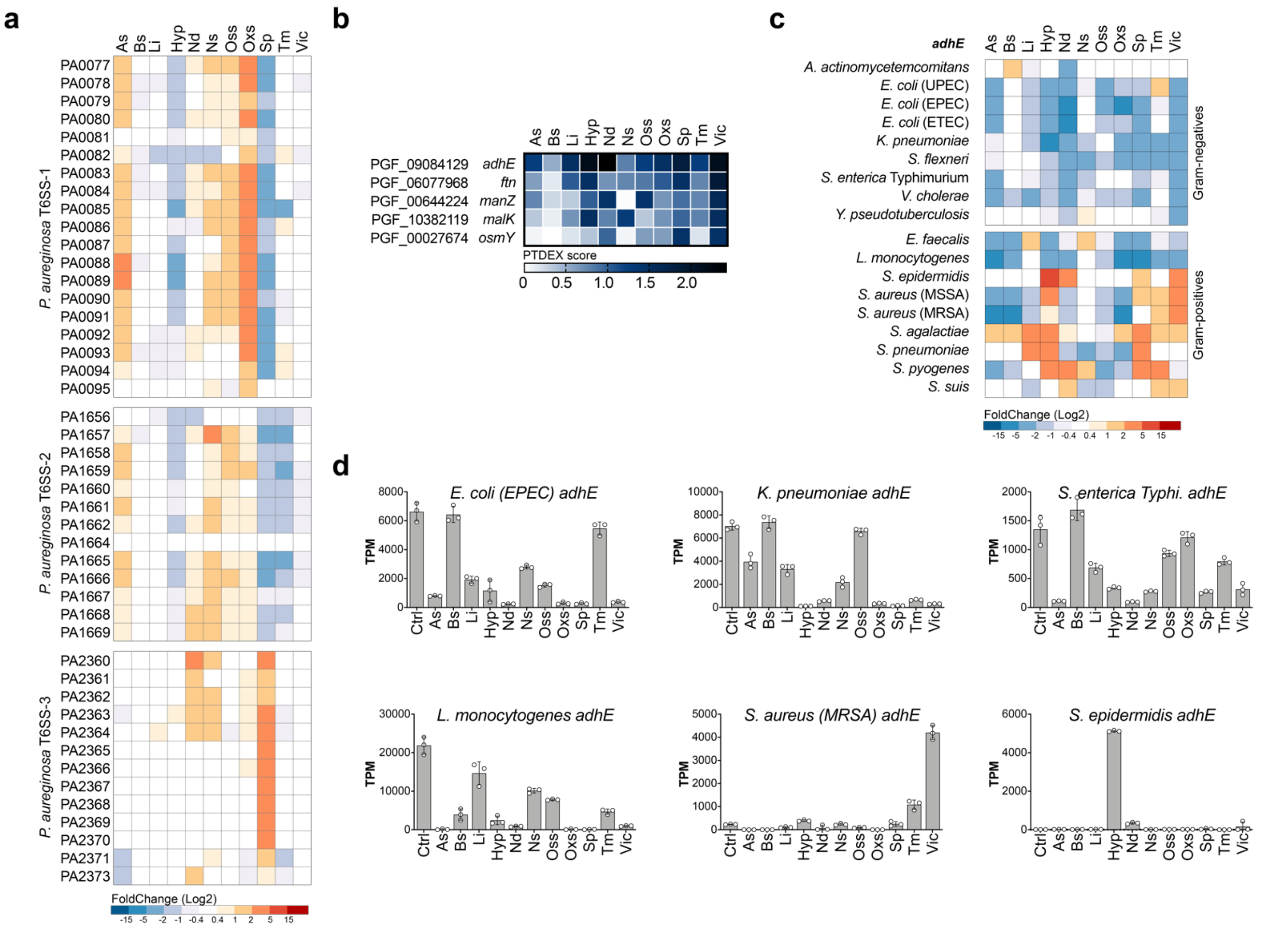

Supplementary Fig. 9 PATHOgenex RNA atlas provides opportunities for retrieval of gene expression data including differential expression values, PTDEX scores and gene expression values. a, Differential expression of the three T6SS operons in *P. aeruginosa* PAO1 under 11 stress conditions. **b,** Top 5 PGFam groups with highest PTDEX scores in virulence inducing condition. **c,** Differential expression of *adhE* in all strains carrying the gene. **d,** Expression levels (TPM) of *adhE* across all conditions in three strains representative of Gram-negative and -positive strains. n=3 independent biological samples from each condition were examined. Data are presented as mean values +/- SD. The differential expression in **a** and **c** are shown as log2 fold change with FDR-corrected *p-value* < 0.05.

Supplementary Table 1. Oligonucleotides used in this study.

Oligonucleotides	Sequence
AR2 primer for cDNA synthesis	TACACGACGCTCTTCCGAT
3Tr3 adapter (3’ linker)	AGATCGGAAGAGCACACGTCTG
2P_univP5 forward primer	AATGATACGGCGACCACCGAGATCTACACTCT TTCCCTACACGACGCTCTTCCGATCT
DNA barcoded adaptors with 5’P and 3’ C3 spacer used for Ctrl_1	AACATTATTAGATCGGAAGAGCGTCGTGTA
DNA barcoded adaptors with 5’P and 3’ C3 spacer used for Ctrl_2	AACCCATGTAGATCGGAAGAGCGTCGTGTA
DNA barcoded adaptors with 5’P and 3’ C3 spacer used for Ctrl_3	AAAGTGTTGAGATCGGAAGAGCGTCGTGTA
DNA barcoded adaptors with 5’P and 3’ C3 spacer used for As_1	AAGAATTATAGATCGGAAGAGCGTCGTGTA
DNA barcoded adaptors with 5’P and 3’ C3 spacer used for As_2	AATATGGACAGATCGGAAGAGCGTCGTGTA
DNA barcoded adaptors with 5’P and 3’ C3 spacer used for As_3	AATCACTTGAGATCGGAAGAGCGTCGTGTA
DNA barcoded adaptors with 5’P and 3’ C3 spacer used for Bs_1	ACGGCACTTAGATCGGAAGAGCGTCGTGTA
DNA barcoded adaptors with 5’P and 3’ C3 spacer used for Bs_2	AGGTCCTCTAGATCGGAAGAGCGTCGTGTA
DNA barcoded adaptors with 5’P and 3’ C3 spacer used for Bs_3	ACTCTAACTAGATCGGAAGAGCGTCGTGTA
DNA barcoded adaptors with 5’P and 3’ C3 spacer used for Oxs_1	ATCATCGTGAGATCGGAAGAGCGTCGTGTA
DNA barcoded adaptors with 5’P and 3’ C3 spacer used for Oxs_2	ATCCCGCGGAGATCGGAAGAGCGTCGTGTA
DNA barcoded adaptors with 5’P and 3’ C3 spacer used for Oxs_3	AGTCTGGCGAGATCGGAAGAGCGTCGTGTA
DNA barcoded adaptors with 5’P and 3’ C3 spacer used for Ns_1	AGAGCCATCAGATCGGAAGAGCGTCGTGTA
DNA barcoded adaptors with 5’P and 3’ C3 spacer used for Ns_2	AGTAACTGCAGATCGGAAGAGCGTCGTGTA
DNA barcoded adaptors with 5’P and 3’ C3 spacer used for Ns_3	ACCCTCGGCAGATCGGAAGAGCGTCGTGTA
DNA barcoded adaptors with 5’P and 3’ C3 spacer used for Oss_1	ACAACTCGCAGATCGGAAGAGCGTCGTGTA
DNA barcoded adaptors with 5’P and 3’ C3 spacer used for Oss_2	ACTCGGTACAGATCGGAAGAGCGTCGTGTA
DNA barcoded adaptors with 5’P and 3’ C3 spacer used for Oss_3	AGCAGCCACAGATCGGAAGAGCGTCGTGTA
DNA barcoded adaptors with 5’P and 3’ C3 spacer used for Li_1	AGAACGATTAGATCGGAAGAGCGTCGTGTA
DNA barcoded adaptors with 5’P and 3’ C3 spacer used for Li_2	ACCAAGTCGAGATCGGAAGAGCGTCGTGTA
DNA barcoded adaptors with 5’P and 3’ C3 spacer used for Li_3	ACCCGTCTTAGATCGGAAGAGCGTCGTGTA
DNA barcoded adaptors with 5’P and 3’ C3 spacer used for Tm_1	ACCCTACAGAGATCGGAAGAGCGTCGTGTA
DNA barcoded adaptors with 5’P and 3’ C3 spacer used for Tm_2	ACCGGTACCAGATCGGAAGAGCGTCGTGTA
DNA barcoded adaptors with 5’P and 3’ C3 spacer used for Tm_3	ACGGAGGGCAGATCGGAAGAGCGTCGTGTA
DNA barcoded adaptors with 5’P and 3’ C3 spacer used for Nd_1	ACTGGATCGAGATCGGAAGAGCGTCGTGTA
DNA barcoded adaptors with 5’P and 3’ C3 spacer used for Nd_2	AGCCTGTATAGATCGGAAGAGCGTCGTGTA
DNA barcoded adaptors with 5’P and 3’ C3 spacer used for Nd_3	AGAGATTGTAGATCGGAAGAGCGTCGTGTA
DNA barcoded adaptors with 5’P and 3’ C3 spacer used for Vic_1	AGGCCCAAGAGATCGGAAGAGCGTCGTGTA
DNA barcoded adaptors with 5’P and 3’ C3 spacer used for Vic_2	AGGTCTGGGAGATCGGAAGAGCGTCGTGTA
DNA barcoded adaptors with 5’P and 3’ C3 spacer used for Vic_3	ATACAACATAGATCGGAAGAGCGTCGTGTA
DNA barcoded adaptors with 5’P and 3’ C3 spacer used for Sp_1	ATACAGATGAGATCGGAAGAGCGTCGTGTA
DNA barcoded adaptors with 5’P and 3’ C3 spacer used for Sp_2	ATACCGGCCAGATCGGAAGAGCGTCGTGTA
DNA barcoded adaptors with 5’P and 3’ C3 spacer used for Sp_3	ATAGCTACAAGATCGGAAGAGCGTCGTGTA
DNA barcoded adaptors with 5’P and 3’ C3 spacer used for Hyp_1	ATTACCACGAGATCGGAAGAGCGTCGTGTA
DNA barcoded adaptors with 5’P and 3’ C3 spacer used for Hyp_2	ATGAACCAGAGATCGGAAGAGCGTCGTGTA
DNA barcoded adaptors with 5’P and 3’ C3 spacer used for Hyp_3	ATGGGAGACAGATCGGAAGAGCGTCGTGTA
DNA barcoded adaptors with 5’P and 3’ C3 spacer used for Hyp_2	ATGAACCAGAGATCGGAAGAGCGTCGTGTA
DNA barcoded adaptors with 5’P and 3’ C3 spacer used for Hyp_3	ATGGGAGACAGATCGGAAGAGCGTCGTGTA
