# Supplementary material for: RNA atlas of human bacterial pathogens uncovers stress dynamics linked to infection
Source: Nat Commun. 2021 Jun 2;12:3282. doi: 10.1038/s41467-021-23588-w (PMC8172932; doi:10.1038/s41467-021-23588-w)
Supplement: Supplementary file 3 — Description of Additional Supplementary Files [file 41467_2021_23588_MOESM3_ESM.pdf]

Description of Additional Supplementary Files

File Name: Supplementary Data 1
Description: Detailed growth and stress conditions for each species in PATHOgenex RNA atlas

File Name: Supplementary Data 2
Description: Number of total reads and mapping efficiency for each RNA-seq library

File Name: Supplementary Data 3
Description: The general PTDEX scores of KEGG orthology groups in every stress condition tested

File Name: Supplementary Data 4
Description: The generalPTDEX scores of PGFam gene groups in every stress condition tested

File Name: Supplementary Data 5
Description: Gram negative specific PTDEX scores of PGFams gene groups for each stress condition together with associated modules

File Name: Supplementary Data 6
Description: Gram positive specific PTDEX scores of PGFam gene groups for each stress condition together with associated modules

File Name: Supplementary Data 7
Description: PGfam gene groups with high PTDEX scores in multiple conditions

File Name: Supplementary Data 8

Description: Species-specific USRs. The plus sign indicates differential regulation under linked stress condition

File Name: Supplementary Data 9
Description: *P. aeruginosa* (PAO1) and *S. aureus* strain 6850 differentially expressed genes during infection and their differential expression under different stress conditions

File Name: Supplementary Data 10
Description: Statistical analysis on percentages of reads mapped to non-CDS regions. The table shows the adjusted p-value with one-way ANOVA of comparisons for each stress condition to the control. The analysis was done with three biological replicates. RNA-seq were not performed with conditions indicated as 'N/A'.
