# Supplementary material for: RNA atlas of human bacterial pathogens uncovers stress dynamics linked to infection
Source: Nat Commun. 2021 Jun 2;12:3282. doi: 10.1038/s41467-021-23588-w (PMC8172932; doi:10.1038/s41467-021-23588-w)
Supplement: Supplementary file 14 — Reporting Summary [file 41467_2021_23588_MOESM14_ESM.pdf]

nature research

Corresponding author(s): Kemal Avican, Maria Fällman

Last updated by author(s): Apr 19, 2021

Reporting Summary

Nature Research wishes to improve the reproducibility of the work that we publish. This form provides structure for consistency and transparency in reporting. For further information on Nature Research policies, see our Editorial Policies and the Editorial Policy Checklist.

Statistics

For all statistical analyses, confirm that the following items are present in the figure legend, table legend, main text, or Methods section.

n/a	Confirmed	
☐	☒	The exact sample size (n) for each experimental group/condition, given as a discrete number and unit of measurement
☐	☒	A statement on whether measurements were taken from distinct samples or whether the same sample was measured repeatedly
☐	☒	The statistical test(s) used AND whether they are one- or two-sided<br>*Only common tests should be described solely by name; describe more complex techniques in the Methods section.*
☐	☒	A description of all covariates tested
☐	☒	A description of any assumptions or corrections, such as tests of normality and adjustment for multiple comparisons
☐	☒	A full description of the statistical parameters including central tendency (e.g. means) or other basic estimates (e.g. regression coefficient) AND variation (e.g. standard deviation) or associated estimates of uncertainty (e.g. confidence intervals)
☐	☒	For null hypothesis testing, the test statistic (e.g. F, t, r) with confidence intervals, effect sizes, degrees of freedom and P value noted<br>*Give P values as exact values whenever suitable.*
☒	☐	For Bayesian analysis, information on the choice of priors and Markov chain Monte Carlo settings
☐	☒	For hierarchical and complex designs, identification of the appropriate level for tests and full reporting of outcomes
☐	☒	Estimates of effect sizes (e.g. Cohen's d, Pearson's r), indicating how they were calculated

Our web collection on statistics for biologists contains articles on many of the points above.

Software and code

Policy information about availability of computer code

Data collection	No software or code used for data collection.
Data analysis	R version 3.5.2 system: x86_64, darwin15.6.0 The R Foundation https://www.r-project.org<br>RStudio Version 1.1.463 Open source https://rstudio.com<br>Python Version 3.6.9 Open source https://www.python.org<br>Jupyter notebook Project Jupyter 6.0.1 https://jupyter.org<br>Venn diagram http://bioinformatics.psb.ugent.be/webtools/Venn/<br>CLC Genomics Workbench Version 20.0.2 QIAGEN https://digitalinsights.qiagen.com<br>Prism 8, Version 8.2.0 Graphpad https://www.graphpad.com<br>PhyloT v2 https://phylot.biobyte.de<br>iTol v6 https://itol.embl.de<br>ClustVis (Beta) https://biit.cs.ut.ee/clustvis<br>CemiTool 1.14.1 https://cemitool.sysbio.tools<br>GhostKOALA Version 2.2 KEGG https://www.kegg.jp/ghostkoala/<br>PATRIC 3.6.9 Proteome Comparison Service tool https://www.patricbrc.org/app/SeqComparison<br>corrplot 0.84 https://cran.r-project.org/web/packages/corrplot/vignettes/corrplot-intro.html

For manuscripts utilizing custom algorithms or software that are central to the research but not yet described in published literature, software must be made available to editors and reviewers. We strongly encourage code deposition in a community repository (e.g. GitHub). See the Nature Research guidelines for submitting code & software for further information.

Data

Policy information about availability of data

All manuscripts must include a data availability statement. This statement should provide the following information, where applicable:

- Accession codes, unique identifiers, or web links for publicly available datasets
- A list of figures that have associated raw data
- A description of any restrictions on data availability

- The sequencing reads generated in this study were deposited in GEO with accession number GSE152295 (https://www.ncbi.nlm.nih.gov/geo/query/acc.cgi?acc=GSE152295) and are publicly avaliable.
- Pseudomonas aeruginosa RNA-seq data from lung tissue or pure cultures and Staphylococcus. aureus RNA-seq data from mouse osteomyelitis model and pure culture are available at GEO under accession number GSE119356 (https://www.ncbi.nlm.nih.gov/geo/query/acc.cgi?acc=GSE119356) and at ENA under the accession number PRJEB6003 (http://www.ebi.ac.uk/ena/data/view/PRJEB6003), respectively
- The processed datasets generated in this study is publicly available at www.pathogenex.org
- The raw data generated in this study will be publicly available upon publication of the manuscript.

Field-specific reporting

Please select the one below that is the best fit for your research. If you are not sure, read the appropriate sections before making your selection.

- [x] Life sciences
- [ ] Behavioural & social sciences
- [ ] Ecological, evolutionary & environmental sciences

For a reference copy of the document with all sections, see nature.com/documents/nr-reporting-summary-flat.pdf

Life sciences study design

All studies must disclose on these points even when the disclosure is negative.

Sample size	No sample size calculation was performed. RNA-seq experiments were performed with 3 biological replicates based on the cost, time, and convenience of data collection with enough statistical power. In accordance with that, hierarchical clustering of expression profiles clustered replicates of the same sample together (Supplementary Fig. 1b), suggesting that the number of replicates is sufficient.
Data exclusions	The data from Bile salts treated samples of B. burgdorferi, B. pseudomallei, H. influenzae, H. pylori strains G27 and J99, L. pneumophila and hypoxia and stationary phase samples from S. suis were excluded from the analyses due to low sequencing reads recovery.
Replication	The sequencing of RNA-seq libraries were highly reproducible as the biological replicates of the same sample clustered together as mentioned above. Each RNA-seq library was sequenced once. We did not replicate our findings as we did not generate experimental data rather than the sequencing data.
Randomization	Randomization was not relevant to this study. The collection of bacterial species was based on being human bacterial pathogens belonging to different phylogenetic orders. The bacterial species were allocated in different groups based on aerobic/microaerophilic growth, Gram staining, and phylogenetic orders.
Blinding	No wet lab experiments except RNA-seq library preparations were performed. Therefore, blinding was not relevant for this study. During sample collection and RNA-seq library preparation same experimental setup and methodology was used for all samples. Blinding was not possible for bacterial species allocated in different groups as the groups were predetermined for subsequent analyses.

Reporting for specific materials, systems and methods

We require information from authors about some types of materials, experimental systems and methods used in many studies. Here, indicate whether each material, system or method listed is relevant to your study. If you are not sure if a list item applies to your research, read the appropriate section before selecting a response.

Materials & experimental systems

n/a	Involved in the study
x	Antibodies
x	Eukaryotic cell lines
x	Palaeontology and archaeology
x	Animals and other organisms
x	Human research participants
x	Clinical data
x	Dual use research of concern

Methods

n/a	Involved in the study
x	ChIP-seq
x	Flow cytometry
x	MRI-based neuroimaging
